# Differential biodistribution of intravenously administered endothelial progenitor and cytotoxic T-cells in rat bearing orthotopic human glioma

**DOI:** 10.1186/1471-2342-13-17

**Published:** 2013-06-10

**Authors:** Nadimpalli Ravi S Varma, Adarsh Shankar, Asm Iskander, Branislava Janic, Thaiz Ferraz Borin, Meser M Ali, Ali S Arbab

**Affiliations:** 1 Cellular and Molecular Imaging Laboratory, Radiology, Henry Ford Hospital, Detroit, MI, USA

**Keywords:** Biodistribution, Endothelial Progenitor Cells, Cytotoxic T-cells, SPECT Imaging And Indium-111 Labeling

## Abstract

**Background:**

A major challenge in the development of cell based therapies for glioma is to deliver optimal number of cells (therapeutic dose) to the tumor. Imaging tools such as magnetic resonance imaging (MRI), optical imaging, positron emission tomography (PET) and single-photon emission computed tomography (SPECT) has been used in cell tracking and/or biodistribution studies. In this study, we evaluate the dynamic biodistribution of systemic injected labeled cells [human cord blood derived endothelial progenitor cells (EPCs) and cytotoxic T-cells (CTLs)] in rat glioma model with *in vivo* SPECT imaging.

**Methods:**

Human cord blood EPCs, T-cells and CD14^+^ cells (monocytes/dendritic cells) were isolated using the MidiMACS system. CD14^+^ cells were converted to dendritic cells (DC) and also primed with U251 tumor cell line lysate. T-cells were co-cultured with irradiated primed DCs at 10:1 ratio to make CTLs. Both EPCs and CTLs were labeled with In-111-oxine at 37°C in serum free DMEM media. Glioma bearing animals were randomly assigned into three groups. In-111 labeled cells or In-111 oxine alone were injected through tail vein and SPECT imaging was performed on day 0, 1, and 3. In-111 oxine activity in various organs and tumor area was determined. Histochemical analysis was performed to further confirm the migration and homing of injected cells at the tumor site.

**Results:**

EPCs and CTLs showed an In-111 labeling efficiency of 87.06 ± 7.75% and 70.8 ± 12.9% respectively. Initially cell migration was observed in lung following inravenous administration of In-111 labeled cells and decreased on day 1 and 3, which indicate re-distribution of labeled cells from lung to other organs. Relatively higher In-111 oxine activity was observed in tumor areas at 24 hours in animals received In-111 labeled cells (EPCs or CTLs). Histiological analysis revealed iron positive cells in and around the tumor area in animals that received labeled cells (CTLs and EPCs).

**Conclusion:**

We observed differential biodistribution of In-111-oxine labeled EPCs and CTLs in different organs and intracranial glioma. This study indicates In-111 oxine based SPECT imaging is an effective tool to study the biodistribution of therapeutically important cells.

## Background

Human glioma is an aggressive, malignant brain tumor and cure rate of presently available therapies (surgical resection, radiotherapy, and chemotherapy) are too poor [1,2]. Cell based gene therapies are growing fast as a potential alternative therapies for the glioma [3-5]. Cell based delivery of therapeutic agents offers advantages over regular vector or viral based delivery due to the ability of cells (such as stem cells) to cross tumor blood brain barrier (TBBB) and for extended homing at the tumor site [6-8] following systemic administration. Development and continual improvement of *in vivo* cell tracking methods are important for ongoing efforts to improve the effectiveness of cell-based therapies for glioma treatment [9]. In cell based therapies, it is important to determine optimal effective dose, monitor cell delivery, number of viable cells and homing to improve the therapeutic effect at the tumor site.

Imaging techniques are necessary to monitor the efficiency of cell retention and dose determination in *in vivo* preclinical models. Magnetic resonance imaging (MRI) has been used in animal models for cell tracking with the help of iron-oxide nanoparticles as probes [10,11]. Studies have shown the capability of iron-oxide nanoparticles for non-invasive MRI tracking of cell migration and engraftment [9,10]. Recently dual-modality probes such as radiolabeled iron oxide nanoparticles have been developed and shown the promising results for biodistribution and diagnostic applications. These probes can be detected by two different imaging modalities (SPECT/PET or MRI) at the same time [12]. In addition, these nanoparticles help in generating high sensitivity (SPECT/PET) and high resolution (MRI) images [12]. The main drawback of iron based nanoparticles is their inability to distinguish live from dead cells. In addition, iron-oxide nanoparticles induce hypointensities on T2 or T2*-weighted images, which further hamper to distinguish from hemorrhage. Recently manganese oxide (MnO) based nanoparticles were developed to overcome these limitations, which show high signal intensity on T1-weighted images [13,14]. MnO based nanoparticles have been used to label stem cells and used *in vivo* MRI to track stem cell migration and engraftment [13,14]. However, MRI does not provide the data on whole body biodistribution of labeled cells as well as number of cell accumulation at the target site. Positron emission tomography (PET) has been used in combination with 18 F-fluorodeoxyglucose (18 F-FDG) for cell tracking. However, long term tracking of cells with PET imaging was difficult due to short half-life of the probes [15,16]. SPECT is advantageous to monitor administered cells for relatively longer time and to observe clearance of cells from different organs and homing at the tumor site [10,17]. For SPECT imaging, either Indium-111 (In-111)-oxine or Technetium-99m (Tc-99m) based radiopharmaceuticals (such as Tc-99 m-HMPAO), can be used to label cells. However, In-111-oxine is better suited due to its longer half-life (2.8 days) to monitor migration and homing of intravenously (IV) administered cells to the site of tumor.

In the present study, we described the method of labeling of EPCs and CTLs with In-111-oxine and investigated differential biodistribution of In-111-oxine labeled cells using SPECT imaging in rat glioma models. SPECT scanning helps to study the whole body and organ specific biodistribution of the cells (EPCs and CTLs). Such information would help determining the percent of administered cells reaching target site (tumor area) and further impacts on dose evaluation that improves therapeutic outcome.

## Methods

### Isolation of EPCs

Human cord blood was collected under Henry Ford Health System institutional review board (IRB) approved protocol (3287) [9,17]. AC133^+^ EPCs were isolated using our previously published method [9,17]. Brief, AC133^+^ cells were separated from human cord blood using ficoll gradient centrifugation and further selected using the MidiMACS system (Miltenyi, Auburn, CA) [9,17]. Collected EPCs were maintained in hematopoietic stem cell media at 1 × 10^6^ cells per ml in 5% CO_2_/95% air at 37°C [9,17]. To confirm the purity of the isolated cells, flow-cytometry was done using different AC133^+^ markers [9,17].

### Preparation of Sensitized T-Cells (CTLs)

T-cells and CD14^+^ cells were isolated from human cord blood as described in our previous published method [10]. CD14^+^ cells were converted to immature dendritic cells (DC) [10]. Immature DCs were subjected maturation process and primed with U251 cell lysate according our published method [10]. Matured DCs were tested for markers specific for DCs (CD14, CD86, CD83 and HLA-DR) by flow-cytometry [10]. Sensitization of T-cells was carried out according to our previous works [10]. Brief, cryopreserved T-cells thawed and cultured overnight and then co-cultured with irradiated primed DCs at 10:1 ratio [10]. T-cell proliferation was determined by MTT assay (ATCC) according to manufacturer instructions. After 6 days of co-culture the T-cells were collected by centrifugation and re-suspended in normal saline for further labeling [10].

### In111 oxine labeling of EPCs, and CTLs

Both EPCs and CTLs were labeled with In-111-oxine (Anazao Health Corp, Tampa FL) at 37°C in normal saline. Twenty million either EPCs or CTLs were incubated with 37 MBq of In-111-oxine in 2 ml of serum free DMEM media for 20–25 minutes. After incubation for 20–25 minutes, the cells were centrifuged (1200 rpm for 10 min) and washed twice with PBS. Cell viability and percentage of labeling efficiency was calculated. Rats were administered with 20 × 10^6^ CTLs or 10 × 10^6^ EPCs or In-111 oxine alone for each respective animal and iron-oxide labeling was also used to label small portion of cells (CTLs or EPCs) to detect cell migration to the tumor area using histochemistry [17].

### Animal model

Athymic nude rats (6–8 weeks of age) (Charles River Laboratory, Inc.) were anesthetized using ketamin/xylazine mixture and were placed on a stereotactic head holder [10,17]. The tumor was implanted according to our published method [10,17]. Brief, a hole was drilled after exposing the skull and U251 glioma tumor cells (4 × 10^5^) were injected stepwise followed by withdrawal of syringe [10,17]. After syringe withdrawal, bone wax was used to close the surgical hole and skin was sutured [10,17].

### SPECT imaging and analysis

Glioma bearing nude rats were randomly assigned into three groups; 1) animals receiving an tail vein administration of In-111-labeled EPCs (10 × 10^6^ cells) (n = 6), 2) animal receiving tail vein injection of In-111 labeled CTLs (20 × 10^6^ cells) (n = 4), 3) animal receiving tail vein injection of equal amount of In-111 oxine (n = 6). After tail vein injection of In-111-labeled cells or In-111-oxine alone, SPECT scanning was performed on day 0 (1–3 hours post injection of labeled cells or In-111-oxine), day1 and 3. Once put on appropriate anesthesia using ketamine/xylazine (100/10 mg/kg), animals were scanned with a SPECT imager (PRISM 3000 gamma camera, Picker, USA) [17]. Whole body (neck to tail region) of the animal was scanned followed by separate scan of the head region. After imaging, rats were euthanized and perfused to collect the organs for further analysis [17]. Acquired images were processed using Bioscan software (HiSPECT, Washington DC) and Image J software (NIH, Bethesda MD). The whole body radioactivity on day 0 upon administration of labeled cells or In-111-oxine was used as total administered dose and activity in organs was calculated as percentage of administered dose after decay correction.

### Organ specific radioactivity and histochemistry

After day 3 SPECT imaging, animals were euthanized using pentobarbital (150–200 mg/kg) and perfused with 3% paraformaldehyde. Tissue samples from heart, lung, kidney, liver and spleen and brain were obtained. The radioactivity was measured for all tissue samples using gamma counter (Wizard 1420, PerkinElmer, USA).

Tissue sections from brain were prepared according to our previous publications [10,17]. In brief, brain samples were made into 1-mm blocks and paraffin embedded. The embedded blocks were cut into serial 10 μm sections and prussian blue staining was used to visualize the iron within FePro labeled cells. Brain tissue sections were stained with prussian blue according to our published works to detect the migration of administered labeled cells to the tumor area [10,11,17]. Brief, fixed sections were de-paraffinized, rehydrated, washed, and incubated for 30 min with 5% potassium ferrocyanide in 5% hydrochloric acid, DAB enhanced, and counterstained with nuclear fast red. Prussian blue stained sections were observed under light microscope to determine the migration of labeled cells (EPCs or CTLs) to tumor area [10,11,17].

### Statistical analysis

The Student *t* test was used to evaluate differences between rat groups, with *P* < 0.05 considered to be statistically significant. All data was expressed as mean ± standard deviation (± SD) unless stated otherwise.

## Results

### Labeling efficiency and cellular viability

EPCs and CTLs were labeled with In-111 oxine under suspension conditions in normal saline. EPCs and CTLs showed a labeling efficiency of 87.06 ± 7.75% and 70.8 ± 12.9% respectively. To test whether labeling with In-111-oxine have any effect on viability of cells, we used trypan blue dye exclusion test to measure the cell viability of labeled and unlabeled CTLs and EPCs. We tested the cell viability immediately after cells labeled with In-111-oxine. Both CTLs and EPCs showed similar viability (no significant differences) following In-111-oxine labeling compared to that of corresponding unlabeled CTLs or EPCs.

### Biodistribution of In-111 labeled cells in different organs

**Figure 1:**
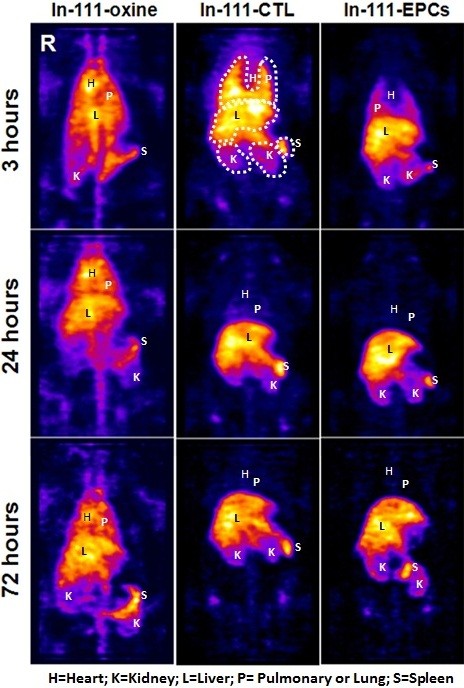
**SPECT images showing biodistribution of IV administered In-111-oxine, In-111 labeled CTLs and EPCs: Biodistribution of In-111-oxine alone did not showed any difference from 3 to 72 hours, but activity in spleen increased at 72 hours.** Labeled CTLs showed high activity in lung at 3 hours and redistribution at 24 and 72 hours. The activity of In-111-CTLs in liver increased from 3 hrs to 72 hours while activity in spleen remained constant. Labeled EPCs showed activity in lung at 3 hours, which is much lower when compared to CTLs. The activity of In-111-EPCs in liver and spleen did not change much with the time (3 to 72 hours).

In-111- CTLs, In-111-EPCs or In-111-oxine were injected IV into glioma bearing nude rats. Biodistribution of radioactivity in different organs and in tumors were determined on days 0, 1 and 3 using SPECT imaging. In-111 oxine injected animal showed identical activity from 3 to 72 hours in lung, heart, brain, and liver, while in spleen an increased activity was observed at 72 hours (Figure 1). We observed differences in biodistribution between CTLs and EPCs, In-111 labeled CTLs showed higher activity in lung at 3 hours scan compared to In-111 labeled EPCs (Figure 1). However, 24 hours scanning showed clearance of activity from the lung, which indicated redistribution of In-111-CTLs to other organs. On the other hand, we observed less activity of In-111-EPCs in the lung at all time points compared to In-111-CTLs. Distribution of cell-associated activities (both CTLs and EPCs) in different organs was clearly different than that of activities seen in animals which received In-111-oxine alone.

**Figure 2:**
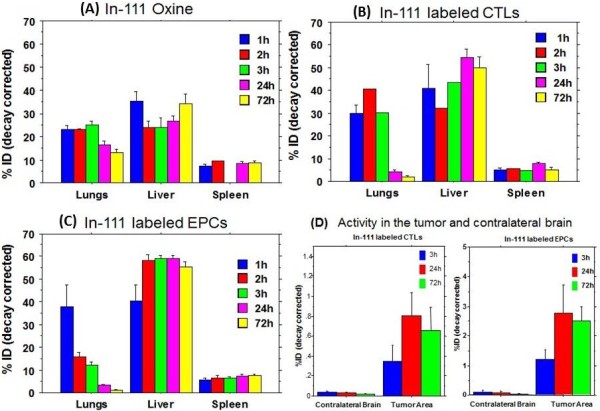
**Graphical representation of percentage of activity in different organs with the time.** Biodistribution study of IV injected In-111 oxine alone **(A)**, In-111 labeled CTLs **(B)**, In-111 labeled EPCs **(C)** and accumulation of In-111-CTLs and In-111-EPCs in glioma **(D)**. In these graphs we included 1 and 2 hours san data along with 3, 24 and 72 hours. **(A)** In-111-oxine alone did not show significant change in organ distribution patterns from 1 to 72 hours. **(B)** Labeled CTLs showed 40% activity in lung at 2 hours, which dropped to 30% at 3 hours and further dropped after 24 and 72 hours, which indicates redistribution for CTLs from lung. Liver and spleen did not showed difference from 1 to 72 hour scans. **(C)** In-111 labeled EPCs showed lung associated activity at 1 hour scan but within 3 hours the activity was dropped by 68.4%, which indicates rapid redistribution of EPCs from the lung. Liver and spleen did not showed significant difference in activity from 3 to 72 hours. **(D)** Percentage of In-111 labeled CTLs and EPCs’ migration to tumor site and contralateral brain was determined in this graph at three time points (3, 24 and 72 hours). CTLs showed highest activity at 24 hours (0.8 ± 0.41%) compared to 3 and 72 hours. EPCs showed highest activity at 24 hours (2.77 ± 2.3%) compared to 3 and 72 hours.

We calculated the percentage of injected dose in liver, lung and spleen following IV administration of In-111-oxine. We observed 23.05 ± 3.22%ID in the lung at 1 hour, which gradually increased to 25.16 ± 2.74%ID at 3 hours but the activity significantly decreased at 24 hours (16.38 ± 3.43%ID) and 72 hours (13.28 ± 2.56%ID) (Figure 2A). However, we did not observe similar shift of radioactivity in the liver. The percentage of radioactivity was not decreased over time and there was no significant difference of In-111-oxine activity among different time points (Figure 2A). Spleen showed significantly less activity of In-111-oxine compared to lung and liver and similar to liver there was no change in activity over time.

We observed initial accumulation of In-111 labeled CTLs in the lung (29.90 ± 4.90%ID at 1 hour to 30.18 ± 0.00%ID at 3 hours) but activity in lung was dramatically decreased at later time point (4.07-2.34%ID at 24 hours), which was further decreased at 72 hours (1.85 ± 1.31%ID) (Figure 2B). However, In-111-CTLs activity in the liver showed gradual increased at 24 hours and 72 hours, which became significantly different compared to that of 2 hours (Figure 2B), while activity in spleen showed no significant differences among the different time points (Figure 2B).

We observed different patterns of distribution for In-111 labeled EPCs in lung compared to that of In-111-CTLs or In-111-oxine. The accumulated In-111-EPCs in the lung (38.02 ± 16.34%ID at 1 hour) started redistribution as early as 2 hours which became significantly lower at 3 hours, which further reduced at 24 and 72 hours (1.13 ± 0.49%ID at 72 hours) (Figure 2C). Early redistribution from the lung was also supported by the increased activity in the liver and spleen at 2 hours. Both the activities in the liver and spleen remained stable until 72 hours (55.55 ± 5.87%ID in liver and 7.5 ± 1.94%ID in spleen) (Figure 2C).

**Figure 3:**
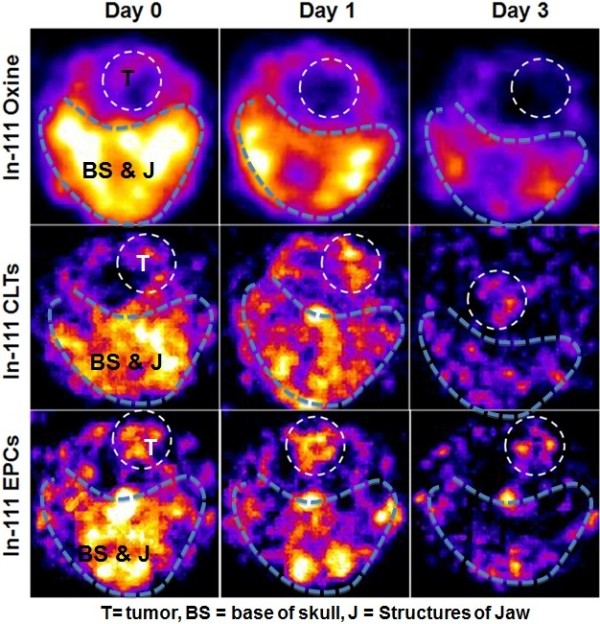
**SPECT images of biodistribution of labeled cells in brain region.** In-111 oxine alone did not show any activity around the tumor area. Accumulation of In-111 labeled CTLs in tumor area was seen within three hours and the activity further increased on 24 hour scans. In-111 labeled EPCs showed high activity around the tumor area in 3 hours scan, which was significantly higher compared to CTLs. 24 hours scan showed further increase in the radioactivity. Note: The increased accumulation of cells after 24 hours of injection. Window levels of signals are kept identical for all day for respective tumors.

We further analyzed migration of injected EPCs and CTLs to the tumor area, where, we observed migration of In-111-CTLs and EPCs to tumor site (Figures 2D and 3). Both In-111-CTLs and In-111-EPCs showed migration to tumors as early as 3 hours post injection. Comparatively higher activity was observed in tumors at 24 hours of post IV administration of In-111-CTLs or In-111-EPCs (Figures 2D and 3). Semi-quantitative analysis showed tumor specific accumulation of CTLs (0.8 ± 0.41%ID at 24 hours) and EPCs (2.7 ± 2.3%ID at 24 hours) (Figure 2D). There was almost negligible activity observed on contra-lateral brain for both CTLs and EPCs.

To further confirm the SPECT image results, we isolated organs (brain, liver, spleen, kidney, heart and lung) after day 3 SPECT scanning and measured the radioactivity *in vitro* using a gamma counter. We found out that radioactivity in the isolated organs are consistent with *in vivo* SPECT imaging data.

### Histochemistry

**Figure 4:**
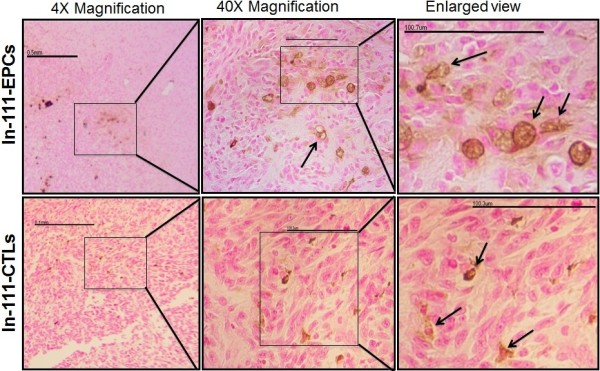
**Histological analysis of brain tumors that received CTLs and EPCs.** DAB enhanced prussian blue staining show multiple iron positive cells in tumors that received CTLs or EPCs (arrows), which indicates migration and homing of CTLs and EPCs into tumor area. 4× magnification was used to show the overview of the brain section with tumor region. 40× magnification was used to show the migration of iron labeled CTLs and EPCs to the tumor area. We also enlarged 40× images to show the intact cells.

Histochemistry based approach was used to further confirm the migration of CTLs and EPCs to the tumor area. Brain sections were prepared and analyzed by staining to determine EPCs and CTLs migration to tumors. We observed iron positive cells in and around the tumor area in animals that received iron oxides nanoparticles labeled CTLs and EPCs (Figure 4). Histochemistry results further support the SPECT imaging findings of EPCs and CTLs migration to the tumor area.

## Discussion

Endothelial progenitor cells and CTLs have been shown to migrate and home to implanted glioma [10,17]. In our previous studies, we showed cord blood derived T-cells can be sensitized against U-251 glioma cells and can be labeled with iron oxide to use as MRI probes to detect glioma from radiation necrosis in rat glioma model [10]. Recently, we showed human cord blood derived EPCs homing at glioma in rat models, where we used iron oxide probes to label the EPCs and tracked them using MRI [11,17]. MRI imaging has limitations to determine the number of migrated labeled cells to the site of interest and whole body biodistribution (different organs) in animal using current set up of hardware and software. To improve the applicability of cell based therapy in cancers and to design effective therapeutic dose, it is important to determine their biodistribution and the rate of migration to tumor site following systemic administration. In this study, we used SPECT imaging in combination with In-111-oxine labeling to determine distribution of administered cells in whole body and in rat model of glioma.

In-111-oxine is commercially available FDA approved radioactive tracer, generally safe to use in humans [18,19]. In-111-oxine widely used in cell labeling due to its lipophilic nature, which passively diffuses into cells and binds to intracytoplamic proteins [20,21]. In addition, half-life of In-111 is 2.8 days, which allows monitoring of cell biodistribution for long period of time (0 to 7 days) in animal models [22]. Before applying the labeled cells in biodistribution study, it is important to know the labeling efficiency and radio toxicity effects on the viability of the cells. The labeling efficiency of In-111-oxine was calculated for the CTLs and EPCs. EPCs showed higher labeling efficiency than that of CTLs. It was not unexpected that In-111-oxine cell labeling efficiency varies with cell type [23]. Several studies showed that In111-oxine labeling of human stem cells did not affect the cell viability and stem cell characteristics [24,25]. Our results with trypan blue dye exclusion assay for cell viability also showed no differences in the viability following In-111-oxine labeling when compared with unlabeled cells. These results indicate that In-111 oxine labeling of CTLs and EPCs did not affect cell viability with the applied dose. These results are also in agreement with previously published report [22].

In our previous studies, we have shown the migration and accumulation of iron nanoparticles labeled CTLs and EPCs to the sites of intracranial implanted glioma in rat models by MRI [10,11]. However, MRI could not determine the biodistribution of IV administered cells in other organs. Although relative number of accumulated cells in the tumor can be predicted by MRI, absolute quantification of (%ID) the number of cell in the area of interest is yet to be determined by MRI. We also used SPECT analysis to determine the migration and accumulation of genetically altered EPCs where cord blood derived EPCs transduced to carry human sodium iodide symporter (hNIS) gene and injected into glioma bearing rats [17]. The accumulation of genetically altered EPCs to the sites of glioma was detected by Tc-99 m-SPECT. We could detect the accumulation EPCs at tumor site but we were not able to determine the number of accumulated EPCs. In this present study, whole body as well as focal SPECT imaging revealed redistribution of injected cells from the different organs (especially from lung). In-111 labeling techniques allowed us to determine the number of accumulated cells (%ID) in different organs as well as in the tumors. Based on the percent ID, we determined the number of accumulated EPCs or CTLs (around 200,000) at 24 hours. We observed an early migration and homing of labeled CTLs and EPCs at tumor site (within 3 hours of injection). This early migration of cells indicates that both CTLs and EPCs have high affinity and early homing properties to the glioma site. The migration of EPCs to tumor site might be due to expression of several factors in tumor cells, predominately expression of SDF-1 or RANTES, which enhance the migration of EPCs and CTLs to tumor site [17]. Accumulation of CTLs and EPCs at tumor site was increased significantly at 24 hours of post injection. This is due to redistribution of CTLs and EPCs from the lung and other organs to the tumor site.

Biodistribution studies of CTLs and EPCs are critical requirement before applying them in clinical studies. In this study we observed migration patterns to lung, liver, and spleen, upon IV injection of CTLs and EPCs. We observed cells migration to lung within hour of systemic injection. Normally, systemically administered cells quickly reach to the heart and travel through pulmonary vasculatures. We observed that cell associated signal from lung was higher at early time points upon systemic injection of CTLs and EPCs. Compared to other organs, lung have microvasculature, which has potential to trap injected cells based on their morphology and adhesion properties [23]. Cells like EPCs can be cleared from lung quickly compared to other cell types such as mesenchymal stem cells (MSCs), which showed higher accumulation and longer retention before redistribution into inflammation sites [23]. We observed reduction of activity from 38% to 3% in lung within 24 hours of post injection of EPCs, which was further reduced after 72 hours. These results indicated that EPCs and CTLs were redistributed faster rate from lung might be due to their smaller size. In addition, we observed an increased activity around the tumor area after 24 hours post injection, which further confirms redistribution of EPCs and CTLs from lung to tumor area. We also observed redistribution of EPCs, which was much faster than CTLs. EPCs showed a rapid clearance from the lung (38% to 12% in 3 hours), while CTLs showed stable activity up to 3 hours (30%ID). This observation might be due to the natural phenomenon of EPCs to migrate quickly to the sites of lesions [26]. On other hand, T-cell upon sensitization with DC begin macromolecular synthesis and enlargement of cytoplasmic volume and the cells size could reaches up to 10–30 μm in diameter [27]. This might be the reason for slow rate of clearance of CTLs from lung.

In this study, we did not evaluate any therapeutic effect of administered cells (CTLs, EPCs) on the glioma. The study was designed to evaluate degree of cell retention in different organs following IV administration, biodistribution patterns, and dose design. The use of the SPECT imaging method in combination with In-111-oxine has been validated as an effective means of cell tracking method to study the biodistribution of administered cells. The data generated in this study can be used to study the timing of injection as well as number of cells accumulation at tumor site. This study also allows calculating the timing for secondary therapeutic dose and retention strategies. In addition, information generated in this study further helps to utilize these cells to deliver therapeutic agents to glioma.

## Conclusion

SPECT studies were able to show the differential bio-distribution of In-111-oxine, EPCs and CTLs in different organs and intracranial glioma. Distribution of In-111-oxine alone in different organs remained identical from day 0 to day 3 except slight increased activity in spleen. Both CLTs and EPCs redistributed from lung to other organs within 24 hours.

## Competing interests

The authors have declared that no competing interests exist.

## Authors’ contributions

NRSV and ASA carried out the experimental design, data analysis and manuscript writing. NRSV carried out the tumour model development and SPECT imaging. NRSV and ASA carried out cell labelling. NRSV, ASA, MMA, BJ carried out image analysis. NRSV, ASMI, AS, TFB carried out histological staining and analysis. All authors read and approved the final manuscript.

## Pre-publication history

The pre-publication history for this paper can be accessed here:

http://www.biomedcentral.com/1471-2342/13/17/prepub
